# Sleep Loss and the Inflammatory Response in Mice Under Chronic Environmental Circadian Disruption

**DOI:** 10.1371/journal.pone.0063752

**Published:** 2013-05-17

**Authors:** Allison J. Brager, J. Christopher Ehlen, Oscar Castanon-Cervantes, Divya Natarajan, Patrick Delisser, Alec J. Davidson, Ketema N. Paul

**Affiliations:** Department of Neurobiology, Morehouse School of Medicine, Atlanta, Georgia, United States of America; Simon Fraser University, Canada

## Abstract

Shift work and trans-time zone travel lead to insufficient sleep and numerous pathologies. Here, we examined sleep/wake dynamics during chronic exposure to environmental circadian disruption (ECD), and if chronic partial sleep loss associated with ECD influences the induction of shift-related inflammatory disorder. Sleep and wakefulness were telemetrically recorded across three months of ECD, in which the dark-phase of a light-dark cycle was advanced weekly by 6 h. A three month regimen of ECD caused a temporary reorganization of sleep (NREM and REM) and wake processes across each week, resulting in an approximately 10% net loss of sleep each week relative to baseline levels. A separate group of mice were subjected to ECD or a regimen of imposed wakefulness (IW) aimed to mimic sleep amounts under ECD for one month. Fos-immunoreactivity (IR) was quantified in sleep-wake regulatory areas: the nucleus accumbens (NAc), basal forebrain (BF), and medial preoptic area (MnPO). To assess the inflammatory response, trunk blood was treated with lipopolysaccharide (LPS) and subsequent release of IL-6 was measured. Fos-IR was greatest in the NAc, BF, and MnPO of mice subjected to IW. The inflammatory response to LPS was elevated in mice subjected to ECD, but not mice subjected to IW. Thus, the net sleep loss that occurs under ECD is not associated with a pathological immune response.

## Introduction

Shift work, travel across time zones, and nontraditional lighting schedules are associated with chronic misalignment of sleep schedules with internal time-keeping systems [1], [2]. These maladaptive work-sleep schedules have escalated across the past decade [3], [4], and have been linked to elevated risks for insomnia and circadian rhythm disorders [5]. Human studies have characterized objective changes in sleep and wakefulness using polysomnography under shorter (<24 h) and longer (>24 h) light-dark (LD) cycles [6], [7], however, few studies have examined sleep during re-entrainment to a shifted LD cycle. In rodents, there exists compelling evidence for the ability of circadian misalignment to alter sleep processes; for example, shorter LD cycles alter the period and amplitude of circadian rhythms, as well as amounts of wake, NREM, and REM sleep [8], [9]. Furthermore, phase shifts of the LD cycle have been shown to split locomotor activity rhythms [10], cause a loss of nocturnality [11], and enhance REM sleep [12]. However, few studies in rodents have explored changes in sleep and wakefulness during exposure to ECD paradigms lasting for more than one month, which are most relevant to long-term night and rotating shift work [5].

The immune system appears to be a target of circadian misalignment and insufficient sleep in both humans [13], [14], [15] and rodents [16], [17], [18]. In a recent study, we reported that circadian disruption in healthy adult mice lasting for one month caused pathological changes in the innate immune response to the bacterial product lipopolysaccharide (LPS; [12]), a condition termed Shift related Inflammatory Disorder (SRID). ECD models the periodic exposure to circadian misalignment experienced by night and rotating shift workers. While we failed to observe sleep loss in a limited polysomnographic study of mice under that regimen of ECD, it is still unknown whether SRID is a consequence of circadian misalignment, sleep loss, or both. Thus, the current study has two aims; first, we report a detailed analysis of sleep/wake dynamics, including changes in amounts, temporal distributions, and rates of re-entrainment of sleep and wake processes, across three months of ECD. Second, we demonstrate that the substantial chronic partial sleep loss associated with this regimen of ECD does not account for the induction of SRID.

## Materials and Methods

### Animals

Adult (4–8 mo of age) *Per2* ^Luc^ knock-in mice bred on a C57BL/6J background (for 12 weeks under ECD) and C57BL/6J mice (for 4 weeks under ECD or imposed wakefulness [IW]) were singly housed in polycarbonate cages at Morehouse School of Medicine. Mice were maintained on a 12 h light:12 h dark (LD) photoperiod at a light intensity of 270 lux from birth in a temperature-controlled vivarium (23°C) with food and water provided *ad libitum.* The experiments complied with *National Institutes of Health Guidelines for the Care and Use of Laboratory Animals* and were approved by the Morehouse School of Medicine Institutional Animal Care and Use Committee.

### Environmental Circadian Disruption

**Figure 1:**
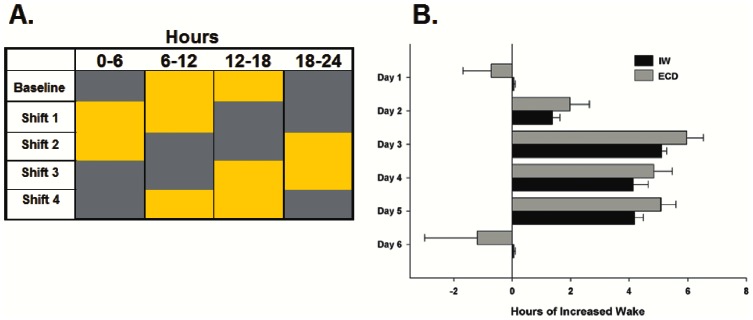
Regimens of environmental circadian disruption (ECD) and imposed wakefulness (IW) were used to examine sleep/wake dynamics during re-entrainment to a phase-advance in the 12 h: 12 h light:dark (LD) cycle and to determine the influences of circadian misalignment and sleep loss on inflammatory markers. *[Left panel; A]* Under ECD, the dark phase (grey area) of LD was phase-advanced by 6 h on the first day of each week for 4 weeks. *[Right panel; B]* The target wake amount under IW was determined from the measurement of wakefulness across 12 weeks of ECD. A two-phase experimental approach that accounted for recovery sleep following long-term periods of imposed wakefulness was undertaken to accurately re-create the amount of sleep loss that occurred under ECD (n = 4). Means±SE of hours of increased wake across each day under IW and ECD.

This protocol is described in [12] and represented in **Fig. 1A**. Briefly, the LD cycle was advanced by 6 h every week for 12 weeks (only the 1st 4 weeks are shown in **Fig. 1A**). Each weekly shift was accomplished by advancing lights-on 6 h during the dark-phase of LD–resulting in one short (6 h) night per week. A separate group of control mice were maintained on a constant LD cycle across 12 weeks to control for any effect of aging [19], [20]. After week 12, all mice were released to constant darkness to characterize sleep/wake dynamics in the absence of photic entrainment across three days.

### Recording and Data Analyses of Sleep and Wakefulness Under ECD

To measure daily changes in sleep and wakefulness across a regimen of ECD, mice received electroencephalograph (EEG) and electromyograph (EMG) electrode implants which were telemetrically interfaced to a data acquisition system (n = 6/treatment group; Dataquest; Data Sciences International; St. Paul, MN). Mice were anesthetized with isoflurane (induced at 2–3%, maintained at 1–2% across surgery). Through a midline incision, two epidural stainless-steel recording screws (SmallParts, Miami Lakes, FL) were positioned contralaterally on the skull (1) 1.0 mm anterior to bregma and 0.5 mm to the right of the central suture; and (2) 0.5 mm posterior to lambda and 1.0 mm to the left of the central suture. A PhysioTel telemeter (model F20-EET; Data Sciences International, St. Paul, MN) was placed in a subcutaneous pocket created caudally along the dorsal surface of the thorax using the existing incision. EMG telemeter leads were bilaterally placed in the nuchal muscle with a 22-gauge needle acting as a trochar, and were sutured to the muscle tissue near the point of entry with 6-0 braided silk. Screw electrodes were then covered with dental acrylic before closing the incision. Post-surgical recovery was 3 weeks during which time the animals received i.p. meloxicam [0.1 ml; 0.5 mg/1.0 ml] injections to manage pain and inflammation. All telemeters were still functional during the 8^th^ week of the study. However, by the beginning of the 12^th^ weekly phase-advance, 3 of the shifted and 4 of the control mice had telemeters with battery power. Therefore, comparisons across shift 12 were conducted separately from those of shifts 4 and 8 (see Text S1).

**Figure 2:**
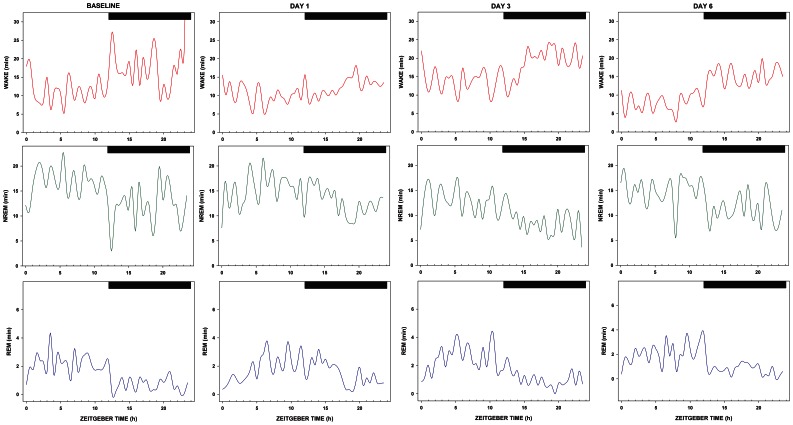
Diurnal distribution of sleep and wake processes across a week under ECD. Mean minutes spent in wake (top), NREM (middle), and REM (bottom) sleep in 30 min blocks across a 24 h recording period at baseline versus the beginning (day 1), middle (day 3), and end (day 6) of week 4 under ECD (n = 6). White-dark bars represent the 12 h light-12 h dark periods of a photocycle with zeitgeber time 12 referring to the light-dark transition.

Polysomnographic waveforms were manually scored in 10-sec epochs using NeuroScore software (Data Sciences International; St. Paul, MN), and were classified as (1) wake (low-amplitude, mixed-frequency EEG; high-amplitude EMG); (2) NREM sleep (high-amplitude, low-frequency EEG; low-amplitude EMG); (3) or REM sleep (low-amplitude EEG with predominant theta activity [6.0–10.0 Hz]; very low-amplitude EMG). Raw waveforms were subjected to Fast Fourier Transform (FFT) to examine EEG power in the delta (0.5–4.0 Hz) range. To determine rates of re-entrainment of sleep and wakefulness across a regimen of ECD, 30 min bins of wake, NREM sleep, and REM sleep across 24 h represented in **Fig. 2** were fitted to sine curves using OriginPro8 (Northhampton, MA; see Text S1). Time points were excluded when the fitted sine curve did not sufficiently match the daily rhythm of sleep and wake (12 time points for shift 4, 13 time points for shift 8).

### Reproduction of Daily Sleep Loss Under ECD

Data from the 12 weeks under ECD were used for a two-phase paradigm of imposed wakefulness (IW) that aimed to mimic amounts of daily sleep loss occurring under 4 weeks of ECD. In order to accurately re-create the amounts of daily sleep loss that occurs under ECD, this paradigm accounted for recovery sleep that follows longer periods of IW.

#### Phase 1

Dose-Response Curve of Daily Sleep Loss. Mice received EEG and EMG implants tethered to a data acquisition system (Pinnacle Technology, Lawrence, KS; n = 4). Sleep and wake were continuously recorded for one week to determine daily time awake during baseline (undisturbed) and during three durations of sle**e**p deprivation induced by a gentle handling procedure (introduction of novel objects into the cage, tapping on the cage and when necessary, delicate touching). This induced sleep deprivation began at lights-on and lasted for 3 h on day 2, 6 h on day 4, and 9 h on day 6. Polysomnographic recordings were manually scored in 10-sec epochs with Sirenia Software (Pinnacle Technology, Lawrence, KS) using criteria described above. The resulting data was used to predict daily amounts of sleep loss that would mirror ECD-induced sleep loss (see **Fig. 1B**).

#### Phase 2

Verification of Predicted Daily Sleep Loss. Mice underwent a four day regimen of imposed wakefulness (IW), accomplished by gentle handling for 1.6 h on day 1, 5.1 h on day 2, 4.2 h on day 3, and 4.3 h on day 4. Total daily sleep loss achieved by the four-day regimen was within 0.8±0.3 h of daily sleep loss on each of the corresponding days of ECD (**Fig. 1B**).

### Post-mortem Validation of Sleep Loss

Fos-immunoreactivity was measured in several sleep-wake regulatory areas of the forebrain (see [23], [24], [25], [26]). Mice were sacrificed on the 7^th^ day (Zeitgeber Time [ZT] 3–4 with ZT 12 representing lights-off) of the 4^th^ week under ECD or IW (n = 8/treatment group). Brains were extracted and immersion-fixed in 4% paraformaldehyde for 24 h followed by immersion in 30% sucrose for 24 h at 4°C. Cryostat sections (40 µm-thick) were incubated with a rabbit polyclonal IgG antibody (c-fos (4); Santa Cruz Biotechnology; Santa Cruz, CA), and Fos expression was visualized using Vectastain Elite ABC kit with 3,3-diaminobenzidine tetrahydrochloride as chromagen (Vector Labs, Burlingame, CA). Sections were mounted with permount, and Fos expression was quantified using ImageJ (National Institutes of Health, Bethesda, MD). Counts of immunostained nuclei were undertaken in the mid-posterior region of the nucleus accumbens (NAc; AP = +1.18 to 0.86), anterior-mid region of the basal forebrain (BF; AP = +0.38 to 0.02), and anterior region of the medial preoptic area (MnPO; AP = +0.38 to 0.26; adapted from [27]).

### Immune Challenge and Cytokine Measurements

In addition to the 12 week ECD schedule, a separate group of mice underwent 4 weeks of ECD. After the 4^th^ shift, these mice were allowed 6 days to re-synchronize their behavioral activity (not shown) to the light-dark cycle. Mice were sacrificed on the 7^th^ day and blood was collected from the trunk under 100% CO2 anesthesia. Using this method, between 500 and 700 µl of blood can be collected from each mouse. At the same time of day (Zeitgeber Time [ZT] 3–4 with ZT 12 representing lights-off), a group of mice that underwent the IW protocol and an unperturbed group of control mice were also sacrificed for blood collection (n = 8/treatment group). Blood was collected in EDTA coated micro tubes. After collection, blood free of clots was diluted five-fold in 1.5 ml micro tubes with RPMI 1640 culture media containing lipopolysaccharide (LPS) to a final concentration of 50 µg/mL. Tubes were tightly closed and were incubated for 3, 6, or 24 h inside a culture incubator kept at 37°C under a 5% CO2 atmosphere. After each incubation time, tubes were removed from the incubator, centrifuged at 1500 g for 10 min, and supernatants were transferred to individual centrifugal filter units (Millipore Corp, Billerica, MA) for concentration according to the manufacturer instructions. Once concentrated, samples were frozen and stored until assayed for interleukin (IL)-6 content using a BD OptIEA ELISA kit (BD, San Diego, CA). For the ELISA assay, all 24 time zero samples were ran on one plate, while the remaining 72 samples (times 3, 6, and 24) were run on a second plate. Both plates were run the same day, using the same freshly prepared standards.

### Statistical Analyses

Repeated measures ANOVAs were used for between- and within-group comparisons of sleep/wake and cytokine parameters. Levels of significance were set at p<0.05. Post-hoc paired t-tests, with Dunn-Sidak correction for multiple comparisons, were used to compare changes in sleep/wake parameters across each day of ECD from baseline levels. Univariate ANOVAs and subsequent Student-Keuls post-hoc tests were used where indicated.

## Results

### Changes in Sleep/Wake under ECD

**Figure 3:**
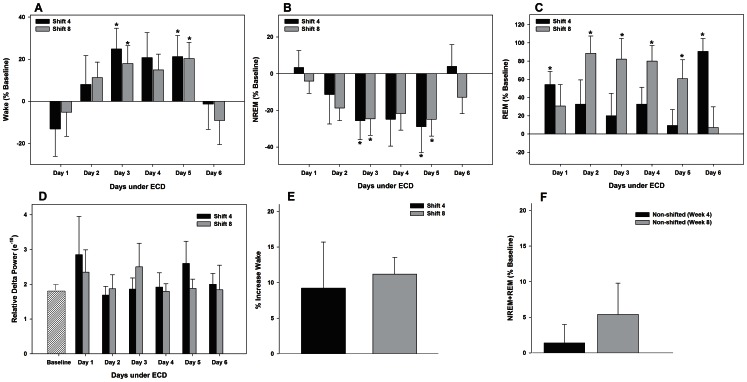
A regimen of ECD maintained for 8 weeks causes temporary reorganzination of daily amounts of sleep and wakefulness. Mean percent change±SE in 24 h amounts of wake (from baseline levels; *A*), NREM sleep (*B*), and REM sleep (*C*) across weeks 4 and 8 of ECD. *D*; The relative delta power of NREM sleep (% power in the delta [0.5–4.0 Hz] frequency range as a percentage of total power [0.5–30.0 Hz]) across baseline and weeks 4 and 8 of ECD; *E*: Mean percent increase±SE in the amount of wake for weeks 4 and 8 under ECD. *F*: Mean percent change±SE in total sleep (NREM+REM) in mice under a non-shifted light-dark cycle for 8 weeks (n = 6). * vs. baseline (post-hoc multiple comparisons by paired t-tests; p<0.004).

There were no between-shift differences in weekly amounts of wake, NREM, and REM sleep (MANOVA main effect of shift; wake F_1,6_ = 1.3, n.s; NREM F_1,6_ = 2.4, n.s.; REM F_1,6_ = 0.1, n.s.; **Table 1**). Significant increases in wake were found on days 3 and 5 for both shifts (vs. baseline day; post-hoc multiple comparisons by paired t-tests, p<0.004; **Fig. 3A**). These increases were at the expense of NREM sleep which was significantly decreased on days 3 and 5 for both shifts (MANOVA main effect of day F_12,60_ = 7.1; p<0.001; post-hoc multiple comparisons by paired t-tests, p<0.004; **Fig. 3B**). REM sleep, however, significantly increased on days 1 and 6 for shift 4 and days 2–5 for shift 8 (MANOVA main effect of day, F_12,60_ = 6.8; p<0.001, post hoc multiple comparisons by paired t-tests, p<0.004; **Fig. 3C**). Relative NREM delta power is used as a measure of sleep intensity and was analyzed across all days of each shift (% power in the delta [0.5–4.0 Hz] frequency range as a percentage of total power [0.5–30.0 Hz]). No differences were found in delta power for either shift (MANOVA main effect of day, F_12,60_ = 0.6; n.s.; **Fig. 3D**). Overall, these changes resulted in decreased total sleep (NREM+REM sleep; MANOVA main effect of day, F_12,60_ = 3.0; p = 0.002; **Fig. 3E**).

**Table 1:** There were no between-shift differences in sleep/wake amounts and fragmentation.

Time (min)	Baseline	Shift 4	Shift 8
WAKE	676.4±26.1	712.0±45.9	710.9±24.2
NREM	694.2±24.5	574.3±.7	557.2±21.2
REM	70.1±5.7	91.9±9.9	104.4±12.3
Bout Number			
NREM	291.1±8.2	353.3±17.4	358.3±23.0
REM	61.5±3.9	94.2±14.4	109.6±17.2
Bout Duration (s)			
NREM	142.0±3.3	97.3±4.5	93.6±5.6
REM	68.4±4.4	73.1±4.5	84.2±1.9
Transitions	794.7±.6	826.6±75.8	832.0±65.4

Week means±SE for shifts 4 and 8. Means±SE for baseline are for a single 24 h recording period.

### Fragmentation

The efficiency (i.e. quality) of sleep can be assessed by the number of sleep episodes (bout number) and the duration of continuous sleep episodes (bout duration). To determine the effects of ECD on sleep efficiency, we examined both NREM and REM bout number and duration. A significant increase in the number of NREM sleep bouts was found on day 3 for shift 4 and day 5 for shift 8 (MANOVA main effect of day, F_12,60_ = 4.4; p<0.001, post hoc multiple comparisons by paired t-tests, p<0.004). A significant increase in the number of REM sleep bouts was found on days 1 and 6 for shift 4 and days 2–5 for shift 8 (MANOVA main effect of day, F_12,60_ = 7.5; p<0.001, post hoc multiple comparisons by paired t-tests, p<0.004). ). A significant decrease in the duration of NREM sleep bouts was found on days 1, 3, and 5 for shift 4 and days 2–5 for shift 8 (MANOVA main effect of day, F_12,60_ = 3.7; p<0.001, post hoc multiple comparisons by paired t-tests, p<0.004). No differences were found in the duration of REM sleep bouts during either shift (MANOVA main effect of day, F_12,60_ = 1.5; n.s.).

### Effects of Aging on Sleep/Wake

In non-shifted animals, total sleep did not change across time (baseline vs. weeks 4 and 8, MANOVA; **Fig. 3F** **;** baseline vs. week 12; **Fig. S1**). As expected, there was a significant effect of ECD on REM sleep compared to non-shifted controls (two-way ANOVA main effect of week, F_1,16_ = 19,1, p<0.001).

### Changes in Fos-Immunoreactivity Under ECD and IW

**Figure 4:**
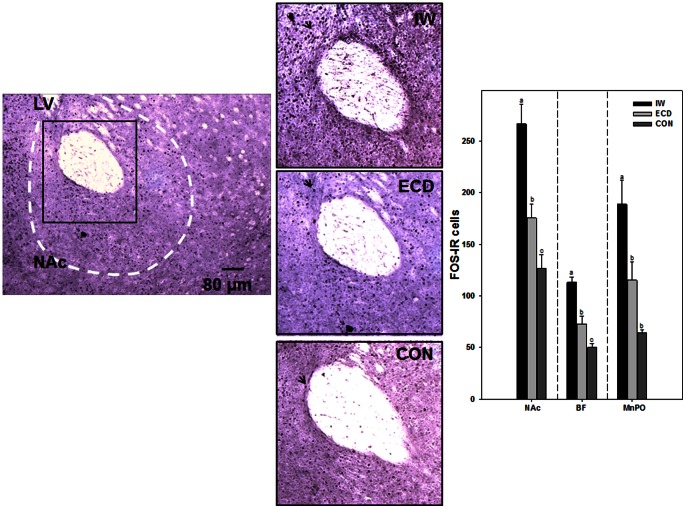
Four week regimens of IW and ECD have differential effects on levels of Fos-immunoreactivity (IR) in sleep-wake regulatory areas of the forebrain. [*Left panel*] Representative photomicrograph of stained Fos-IR nuclei in the nucleus accumbens (NAc). LV; lateral ventricle. [*Middle panel*] Magnified inserts of stained Fos-IR nuclei (arrow) in the NAc of mice that underwent IW, ECD, or were left undisturbed (CON). [*Right panel*] Means±SE of stained Fos-IR nuclei in the NAc, basal forebrain (BF), and medial preoptic area (MnPO; n = 8/group). Bars with different letters are significantly different (p<0.05).

Mice subjected to IW had significantly greater amounts of Fos-IR nuclei in the nucleus accumbens (NAc), basal forebrain (BF), and medial preoptic area (MnPO) compared with mice subjected to ECD or no treatment (CON); NAc: 266.3±19.3 cells (IW) vs. 175.4±13.8 cells (ECD) vs. 126.7±13.4 cells (CON); ANOVA; F_2, 21_ = 20.2; p<0.001, post-hoc; BF: 113.3±4.9 cells vs. 73.1±7.1 cells vs. 50.0±4.1 cells, respectively; ANOVA; F_2, 21_ = 33.6; p<0.001, post-hoc; MnPO: 188.7±23.2 cells vs. 115.3±17.8 cells and 64.2±3.2 cells, respectively; ANOVA; F_2, 21_ = 11.0; p<0.001, post-hoc; **Fig. 4**.

### Changes in Inflammatory Responses After ECD and IW

**Figure 5:**
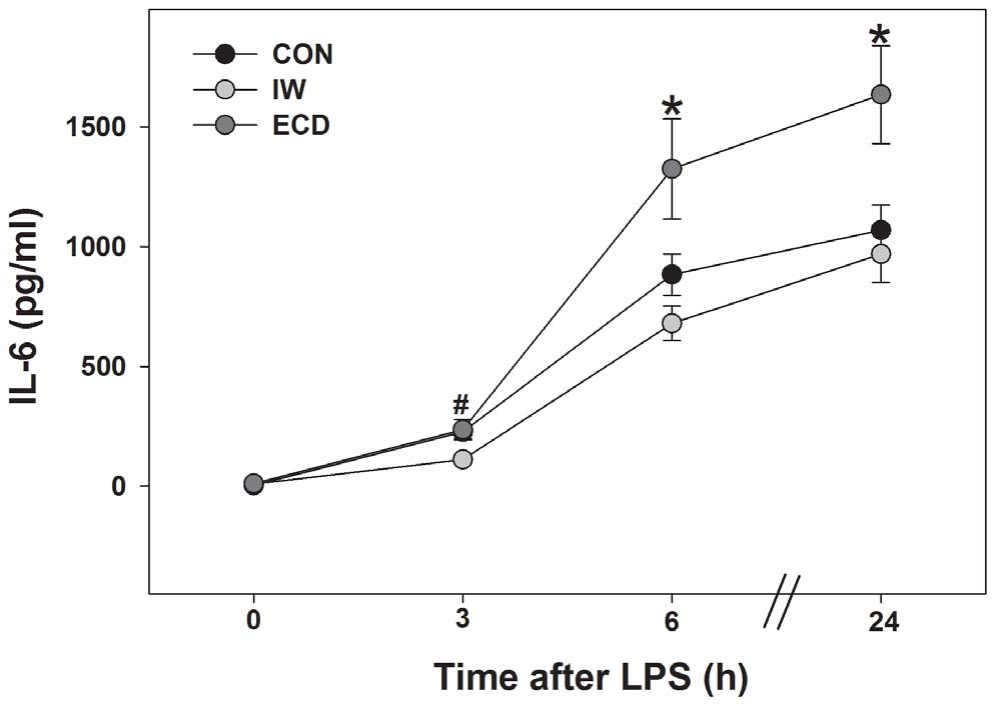
Four weeks of ECD elevate the innate immune response to the bacterial product lipopolysaccharide (LPS). Means±SE of IL-6 content across 24 h of incubation in LPS (n = 8/group). * ECD vs. IW and CON (p<0.05). ^#^ IW vs. ECD and CON (p<0.05).

The time course response to the LPS *ex vivo* challenge in groups of ECD, IW, and control mice is shown in **Fig. 5**. Levels of IL-6 in the ECD group were significantly elevated within 6 h of LPS challenge and remained significant 24 h after LPS (vs. IW and controls, MANOVA main effect of treatment, F_2,21_ = 5.7; p = 0.01).

## Discussion

The present study provides assessments of changes in the amount, fragmentation, and re-entrainment of sleep and wake processes during chronic (1–3 months) exposure to ECD in rodents. We expand upon Castanon-Cervantes *et al.* 2010 [12] by examining daily sleep/wake dynamics across 3 months of ECD. This study also demonstrates the differential effects of circadian misalignment and sleep loss on pro-inflammatory responses in rodents. These data indicate that a substantial, but temporary reorganization or disorganization of sleep and wake processes occurs across each week of a 3 month regimen of phase-advances in the LD cycle. At each week analyzed, a 10–20% reduction in NREM sleep and a 40–100% increase in REM sleep were found. Overall, these changes resulted in an approximately 10% net loss of sleep each week relative to baseline levels, but sleep amounts returned to levels similar to baseline by the end of the week. The largest changes in NREM (decrease) and REM (increase) sleep amounts occurred several days into re-entrainment.

### Sleep/Wake Dynamics Under ECD

The observed changes in amounts of sleep and wake under ECD are consistent with previous studies in humans and animal models. In human studies, shift work and trans time-zone travel has been shown to result in insufficient sleep [5] and physiological sleepiness [21]. It has also been shown that shorter (<24 h) LD cycles increase daily REM sleep amounts by as much as 175% in mice [9]. Interestingly, the percent change in daily REM sleep reported in Deboer *et al.* 2007 [9] is close in magnitude to the 161% increase in daily REM sleep found by the 12^th^ week under ECD in the present study (**Fig. S1**). In both Deboer *et al.* 2007 and the present study, the percent increase in daily REM sleep coincided with a change in REM fragmentation, suggesting that the REM sleep process is sensitive to abrupt and/or repeated changes in the LD cycle. Finally, there were no differences in sleep and wake processes across 3 months in animals maintained on a non-shifted LD cycle indicating that the long duration of the study did not substantially alter sleep-wake architecture.

Abrupt or repeated shifts in the LD cycle also alter sleep efficiency. In the present study, we observed substantial but temporary reorganization of both the duration and number of daily NREM, and REM sleep bouts across the regimen of ECD. These patterns of reorganization included a decrease in the number and duration of NREM bouts and an increase in the number of REM bouts. Despite this change in sleep efficiency, there was no change in relative delta power of NREM sleep implicating that ECD did not alter homeostatic sleep pressure.

Previous studies have also investigated sleep and wake dynamics under shorter or shifted LD cycles. For example, in Cambras *et al.* 2007 [8], housing under a 22 h LD cycle dissociated rhythms of wake, NREM, and REM sleep. Rhythms of wake and NREM sleep both bifurcated, with one rhythm remaining entrained to the LD cycle and the secondary rhythm matching the animals’ free-running periods. Only the rhythm of REM sleep, which matched the animals’ free-running periods, was maintained. Another study found that the acrophases of REM and NREM rhythms increasingly separate across time following a large, phase-delay (8 h) in the LD cycle [22]. In the current study, we investigated the re-entrainment of sleep and wake processes across ECD by fitting a sine curve to the 24 h pattern of wake, NREM, and REM sleep expressed in 30 min bins (**Fig. 2**). The peak 24 h time of wake, NREM, and REM sleep re-entrained 1–2 days after a shift in the LD cycle (**Fig. S2**). This is notable because in most cases, the largest changes in 24 h amounts of wake, NREM, and REM sleep were seen 3–5 days after a shift in the LD cycle. Similar to the study of Lee *et al.* 2009 [22], we also found significant separation in the peaks of REM and NREM sleep by the end of the 4^th^ and 8^th^ week of ECD, providing evidence of circadian disruption. Hence, future studies ought to focus on the mechanism, at the level of the sleep/wake regulatory areas in the brain, to better understand changes in sleep/wake amounts during re-entrainment to chronic circadian disruption.

The dysregulation between arousal- and sleep-promoting areas during night shift work as predicted in Postnova *et al.* 2012 [21] may offer an explanation for our Fos-immunoreactivity data; we found an elevation in Fos-IR in areas of the forebrain regulating arousal and sleep homeostasis, including the nucleus accumbens and basal forebrain, of mice subjected ECD compared with mice left undisturbed. Previous studies have reported elevations in Fos-IR in these areas following acute sleep deprivation [23], [24], [25], [26] as well as an attenuation of Fos-IR in some of these areas during recovery sleep [26]. Few studies, however, have looked at Fos-IR following chronic exposure to sleep deprivation. In this study, we found that mice that had undergone a 4 week regimen of IW aimed to mimic daily sleep amounts under a 4 week regimen of ECD had the greatest amounts of Fos-IR in all areas of the forebrain compared with mice subjected to ECD or left undisturbed. Our Fos-IR data suggest that IW and ECD taxed major arousal-promoting and sleep homeostatic areas of the brain to a different extent despite similar amounts of sleep loss induced by these treatments. Thus, the magnitude of sleep loss manifest from ECD cannot be entirely explained by changes in neuronal activation in sleep-wake circuitry.

### ECD Changes in the Innate Immune Response

These data support our hypothesis that chronic exposure to ECD and not imposed wakefulness accounts for the elevated innate immune response to the bacterial product lipopolysaccharide (LPS), and mirror changes that we have previously reported for an *in vivo* LPS challenge and *in vitro* LPS challenge of macrophages [12]. The results are notable since sleep amounts have been shown to modulate immune responses. For example, sleep disruption has been linked to changes in blood concentrations of immune components such as IL-1β, IL-6, and IFN-γ [28]. However, paradigms used to investigate sleep modulation of the immune response have differed in deprivation methods [28]. Further, chronic sleep disruption concurrent with insufficient recovery is also possible during chronic circadian misalignment as indicated by significant changes in daily sleep/wake amounts across 3 months of ECD in the present study; However, we found that imposed wakefulness in the absence of ECD did not reproduce SRID, suggesting that a history of ECD is necessary for changes in the inflammatory response to LPS response. Future studies should investigate if ECD in the absence of sleep loss or if more subtle sleep alterations associated with ECD contribute to the enhanced immune response to LPS.

### Significance

The present study provides the first assessment of changes in the amount, fragmentation, and re-entrainment of sleep and wake processes during environmental circadian disruption relevant to long-term shift work. This study also recapitulates our previous work showing that sleep loss during exposure to environmental circadian disruption does not account for pathological changes in the innate immune response to the bacterial product lipopolysaccharide (LPS) with circadian misalignment. From a translational perspective, this study demonstrates the vast consequences of shift work or travel across time zones on sleep and inflammatory processes at central and peripheral levels of study.

## Supporting Information

Figure S1 **Significant changes in NREM and REM sleep during the 12^th^ week under environmental circadian disruption (ECD).** Graph shows daily 24 h means±SEM of NREM, and REM sleep across the 12^th^ week under ECD. [Insert] Table shows weekly 24 h means±SEM of NREM and REM sleep in shifted (ECD) versus control (non-shifted) animals. ^a^ shifted vs. control (one-way ANOVA; p<0.05).
(TIF)

Figure S2 **Differential misalignment of sleep/wake processes under environmental circadian disruption (ECD).** [Left Panel] Peak 24 h times of wake, NREM, and REM sleep across each day of the 4^th^ and 8^th^ week under ECD. * vs. baseline (paired t-tests; p<0.05). [Right panel] Peak 24 h time of REM sleep plotted against peak 24 h time of NREM sleep across each day of the 4^th^ week (top) and 8^th^ week (bottom) under ECD. Zeitgeber time 12 refers to the light-dark phase transition in the photocycle. * REM vs. NREM (one-way ANOVA; p<0.05).
(TIF)

Text S1 (DOCX)
