# Supplementary figures and images for: Sleep Loss and the Inflammatory Response in Mice Under Chronic Environmental Circadian Disruption
Source: PLoS One. 2013 May 17;8(5):e63752. doi: 10.1371/journal.pone.0063752 (PMC3656961; doi:10.1371/journal.pone.0063752)

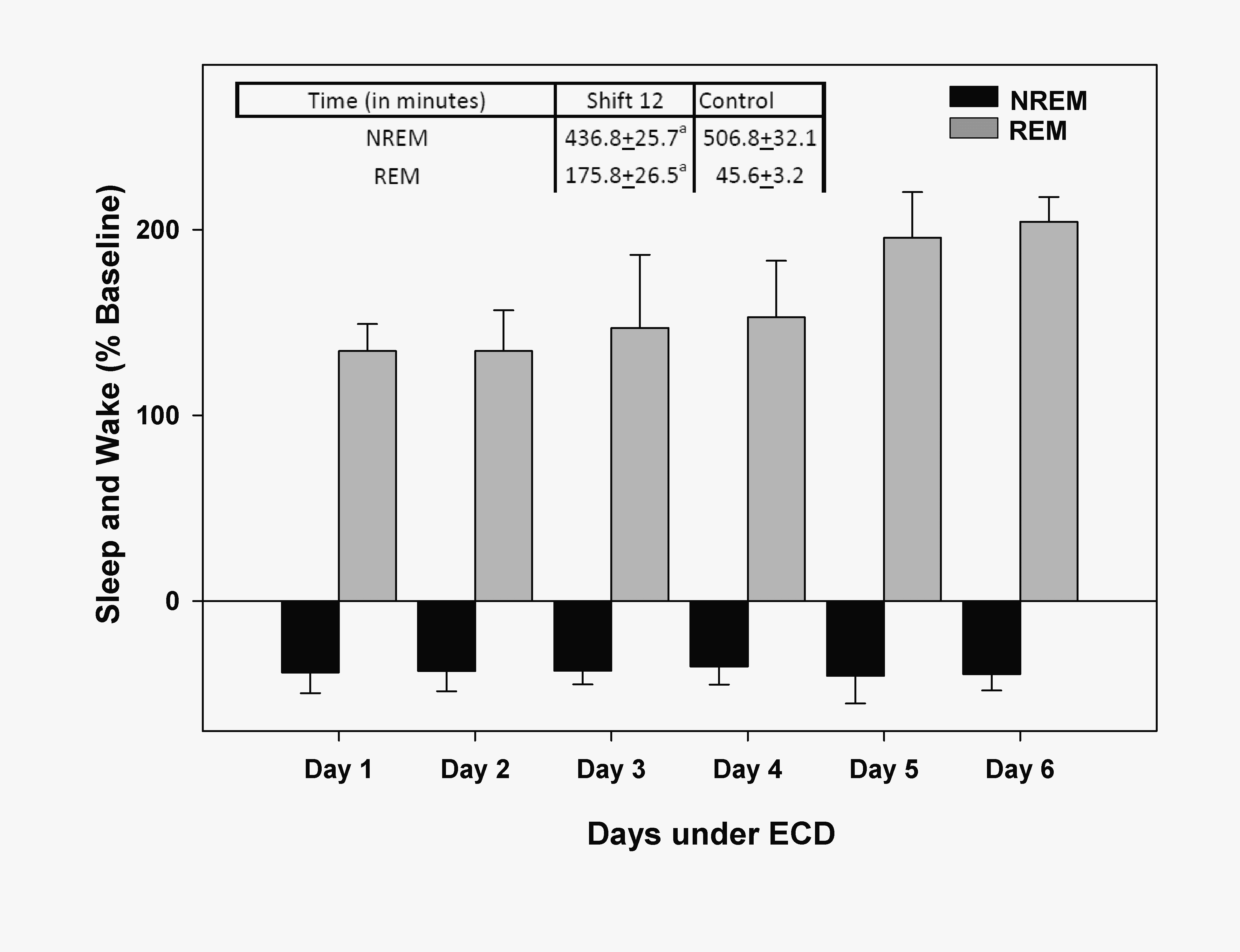

Supplement: Figure S1 — Significant changes in NREM and REM sleep during the 12th week under environmental circadian disruption (ECD). Graph shows daily 24 h means±SEM of NREM, and REM sleep across the 12th week under ECD. [Insert] Table shows weekly 24 h means±SEM of NREM and REM sleep in shifted (ECD) versus control (non-shifted) animals. a shifted vs. control (one-way ANOVA; p<0.05). (TIF) [file pone.0063752.s001.tif]
