# Supplementary material for: Sleep Loss and the Inflammatory Response in Mice Under Chronic Environmental Circadian Disruption
Source: PLoS One. 2013 May 17;8(5):e63752. doi: 10.1371/journal.pone.0063752 (PMC3656961; doi:10.1371/journal.pone.0063752)
Supplement: Text S1 — (DOCX) [file pone.0063752.s003.docx]

**Text S1**

*Changes in Sleep and Wake across 12^th^ Week of Environmental Circadian Disruption (ECD)*

There was no change in total sleep (NREM+REM sleep; MANOVA main effect of day, F_1,5_=1.4; n.s.). There was a significant decrease in NREM sleep (MANOVA main effect of day, F_1,5_=6.8; p=0.003; **Fig. S1**), and a significant increase in REM sleep (MANOVA main effect of day, F_1,5_=4.2; p=0.016; **Fig. S1**). Following release to constant darkness, REM sleep amounts recovered to baseline levels (MANOVA main effect of day, F_1,5_=4.5; n.s.), while NREM sleep amounts remained different from baseline levels (MANOVA main effect of day, F_1,5_=205.6; p<0.001). *Controls.* There was a difference in NREM and REM sleep amounts between mice under ECD and non-shifted (control) mice (one-way ANOVA; NREM: F_1,4_=6.0; REM: F_1,4_=19.1; both, p<0.05).

*Rates of Re-entrainment of Sleep and Wake Processes Across ECD*

There was no between-shift (shift 4 vs. shift 8) difference for peak 24 h time of wake, NREM, and REM sleep (MANOVA main effect for shift; F_1,10_=0.6; p>0.05). Peak 24 h times of wake were delayed by 3.6±0.2 h on day 1 for shift 4 and by 2.3±0.4 h on day 2 for shift 8 (paired t-tests; p<0.05; **Fig. S2**). Peak 24 h times of NREM sleep were delayed by 3.4±0.4 h on day 1 for shift 4 and by 2.2±0.4 h on day 2 for shift 8 (paired t-tests; p<0.05; **Fig. S2**). Peak 24 h times of REM sleep were delayed by 4.1±0.3 h on day 1 for shift 4 and by 2.1±0.4 h on day 2 for shift 8 (paired t-tests; p<0.05; **Fig. S2**). Comparisons of peak 24 h time of REM versus NREM sleep revealed no between-shift difference (MANOVA main effect for shift; F_1,10_=0.9; p>0.05). There was a difference in peak 24 h time of REM versus NREM sleep on day 6 for shift 4 and days 4-6 for shift 8 (one-way ANOVA; all, p<0.05; **Fig. S2**).
